# Comparison of six different CAD/CAM retainers vs. the stainless steel twistflex retainer: an in vitro investigation of survival rate and stability

**DOI:** 10.1007/s00056-023-00486-y

**Published:** 2023-06-28

**Authors:** Christoph J. Roser, Carolien Bauer, Lutz Hodecker, Andreas Zenthöfer, Christopher J. Lux, Stefan Rues

**Affiliations:** 1 Department of Orthodontics and Dentofacial Orthopedics, Heidelberg University Hospital, Im Neuenheimer Feld 400, 69120 Heidelberg, Germany; 2 Department of Prosthodontics, Heidelberg University Hospital, University of Heidelberg, Im Neuenheimer Feld 400, Heidelberg, Germany

**Keywords:** Computer-aided design/computer-aided manufacturing, Orthodontic treatment, Bonded retainer, Breakage, Fixed orthodontic appliances, Computergestütztes Design/computergestützte Fertigung, Kieferorthopädische Behandlung, Gebondeter Retainer, Durchbrechen, Festsitzende kieferorthopädische Apparaturen

## Abstract

**Purpose:**

To compare failure rates and maximum load capacity (F_max_) of six different computer-aided design/computer-aided manufacturing (CAD/CAM) retainers with those of the hand-bent five-stranded stainless steel twistflex retainer.

**Materials and methods:**

Six groups (*n* = 8 per group) of commercially available CAD/CAM retainers (cobalt–chromium [CoCr], titanium grade 5 [Ti5], nickel–titanium [NiTi], zirconia [ZrO_2_], polyetheretherketone [PEEK], and gold) and twistflex retainers were tested for long-term sufficiency and for F_max_ using a self-developed in vitro model. All retainer models underwent a simulated ageing process of about 15 years (1,200,000 chewing cycles with a force magnitude of 65 N at 45° followed by storage in water at 37 °C for 30 days). If retainers did not debond or break during ageing, their F_max_ was determined in a universal testing machine. Data were statistically analysed using Kruskal–Wallis and Mann–Whitney U‑tests.

**Results:**

Twistflex retainers did not fail (0/8) during ageing and had the highest F_max_ (445 N ± 51 N). Ti5 retainers were the only CAD/CAM retainers that also did not fail (0/8) and had similar F_max_ values (374 N ± 62 N). All other CAD/CAM retainers had higher failure rates during ageing and significantly lower F_max_ values (*p* < 0.01; ZrO_2_: 1/8, 168 N ± 52 N; gold: 3/8, 130 N ± 52 N; NiTi: 5/8, 162 N ± 132 N; CoCr: 6/8, 122 N ± 100 N; PEEK: 8/8, 65 ± 0 N). Failure was due to breakage in the NiTi retainers and debonding in all other retainers.

**Conclusion:**

Twistflex retainers remain the gold standard regarding biomechanical properties and long-term sufficiency. Of the CAD/CAM retainers tested, Ti5 retainers seem to be the most suitable alternative. In contrast, all other CAD/CAM retainers investigated in this study showed high failure rates and had significantly lower F_max_ values.

## Abstract (de)

**Zielsetzung:**

Vergleich der Versagensraten und der maximalen Belastbarkeit (F_max_) von 6 unterschiedlichen CAD/CAM(„computer-aided design/computer-aided manufacturing“)-Retainern mit denen eines handgebogenen 5‑strängigen Twistflex-Retainers aus Edelstahl.

**Material und Methoden:**

Sechs Gruppen (*n* = 8 pro Gruppe) kommerziell verfügbarer CAD/CAM-Retainer (Kobalt-Chrom [CoCr], Titan Grade 5 [Ti5], Nickel-Titan [NiTi], Zirkoniumdioxid [ZrO2], Polyetheretherketon [PEEK] und Gold) und Twistflex-Retainer wurden anhand eines selbst entwickelten In-vitro-Modells auf ihre Langzeitstabilität und auf F_max_ getestet. Alle Retainermodelle wurden einem simulierten Alterungsprozess von etwa 15 Jahren unterzogen (1.200.000 Kauzyklen mit einer Kraft von 65 N bei 45 °, gefolgt von einer 30-tägigen Lagerung in Wasser bei 37 °C). Wenn sich die Retainer während der Alterung nicht ablösten oder brachen, wurde ihre F_max_ in einer Universalprüfmaschine bestimmt. Die Daten wurden mit Kruskal-Wallis- und Mann-Whitney-U-Tests statistisch ausgewertet.

**Ergebnisse:**

Twistflex-Retainer versagten während der Alterung nicht (0/8) und hatten die höchste F_max_ (445 N ± 51 N). Ti5-Retainer waren die einzigen CAD/CAM-Retainer, die ebenfalls nicht versagten (0/8) und ähnliche F_max_-Werte aufwiesen (374 N ± 62 N). Alle anderen CAD/CAM-Retainer hatten höhere Versagensquoten während der Alterung und signifikant niedrigere F_max_-Werte (*p* < 0,01; ZrO_2_: 1/8, 168 N ± 52 N; Gold: 3/8, 130 N ± 52 N; NiTi: 5/8, 162 N ± 132 N; CoCr: 6/8, 122 N ± 100 N; PEEK: 8/8, 65 ± 0 N). Das Versagen war bei den NiTi-Retainern auf Brechen, bei allen anderen Retainern auf Debonding zurückzuführen.

**Schlussfolgerung:**

Twistflex-Retainer sind nach wie vor der Goldstandard im Hinblick auf biomechanische Eigenschaften und Langzeitstabilität. Von den getesteten CAD/CAM-Retainern scheinen Ti5-Retainer die am besten geeignete Alternative. Im Gegensatz dazu wiesen alle anderen untersuchten CAD/CAM-Retainer hohe Versagensquoten und deutlich niedrigere F_max_-Werte auf.

## Introduction

Digital techniques are being increasingly integrated into diagnostics and treatment in orthodontics. For example, fully automated digital cephalometric analysis [1] or digital model analysis [2] has been used in diagnostics, and computer-aided design and manufacturing (CAD/CAM) technology has been used to design and fabricate treatment devices. CAD/CAM technology has many applications in orthodontics, including anchoring appliances [3], customised archwires [4] and brackets [5–7], which can be placed intraorally by CAD/CAM-produced bonding trays [8–10]. We recently introduced a CAD/CAM-fabricated functional regulator 3 (CAD-FR3), which extended CAD/CAM application to the production of removable functional appliances [11]. Aligners can also be produced by CAD/CAM, since the different therapy steps and respective models are planned digitally and manufactured using a three-dimensional (3D) printer [12, 13].

Fixed orthodontic CAD/CAM retainers, which are made from various materials including polyetheretherketone (PEEK) [14, 15], nickel–titanium (NiTi) [16–19], and zirconia (ZrO_2_) [20], have emerged as potential alternatives to hand-bent retainers in the last few years. These CAD/CAM retainers have been considered advantageous because of their high-precision fit in demanding situations [21] and because they can be produced automatically by the technician without bending. However, it is important to consider long-term survival rates because retainers can only provide long-term retention if they restrain physiological mastication forces over a long time.

It remains unclear whether CAD/CAM retainers have better long-term survival than hand-bent retainers because studies have only evaluated CAD/CAM retainers for up to one year [17, 19, 22, 23]. This is not sufficient because a retainer may fail years after the orthodontic treatment is finished [24]. Thereby retainer failure can cause complications such as orthodontic relapse [24] or enamel damage caused by the removal of retainers [25]. It is moreover important to note here that complications might not be detected by the orthodontist, since the orthodontic therapy was usually finished years before, and therefore the anterior alignment may relapse. To avoid these complications, it is important to evaluate the long-term survival of different retainers to recommend future treatment.

The aim of the present study was to compare the long-term stability of six novel CAD/CAM retainers with that of the conventional five-stranded twistflex retainer in an in vitro approach. For each tested retainer, the failure rate was recorded during a simulated ageing process and maximal load capacity values (F_max_) were determined. The null hypothesis was that conventional twistflex retainers show inferior performance regarding failure rate and F_max_ compared to CAD/CAM retainers.

## Materials and methods

### Test model for the investigation

**Fig. 1:**
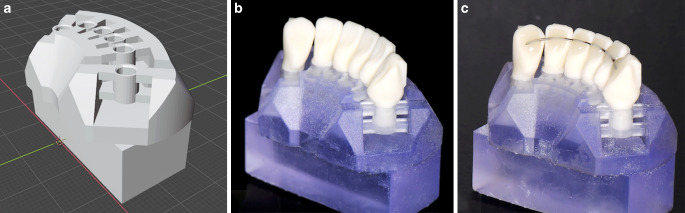
In vitro tooth model. The model base and the teeth were planned and manufactured using computer-aided design/computer-aided manufacturing (CAD/CAM) technology. The model base contained flexible bars that simulated physiological tooth mobility (**a**). All teeth provided planar loading sites on the vestibular surface and were embedded in the model base (**b**). Retainers were bonded on the models (**c**) In-vitro-Zahnmodell. Der Modellsockel und die Zähne wurden mit Hilfe der CAD/CAM(„computer-aided design/computer-aided manufacturing“)-Technologie geplant und hergestellt. Die Modelbasis war mit nachgiebigen Stegen konstruiert, um die physiologische Zahnbeweglichkeit zu simulieren (**a**). Alle Zähne wurden vestibulär und inzisal mit planen Belastungsflächen konstruiert und waren in den Modellsockel eingebettet (**b**). Die Retainer wurden auf die Modelle geklebt (**c**)

For the investigation, a CAD/CAM in vitro model was developed to test the retainers. The model consisted of six artificial teeth (canine to canine) of a lower jaw embedded in a model base (Fig. 1). The model base and the artificial teeth were digitally planned (Geomagic Design X; 3D Systems, Rock Hill, SC, USA) and manufactured separately. The teeth provided planar loading sites on the vestibular surface and were milled from fibre-reinforced composite discs (FRCs; Trinia, Bicon, Boston, MA, USA). The FRCs were tested in advance with the recommended primer (Ceraresin Bond 1&2, Shofu, Tokyo, Japan) and a dental composite (Transbond XT) according to DIN 13990‑1 and had a similar shear bond strength (18.0 ± 2.4 MPa) to that of the clinical situation [26, 27].

The model base was made of resin (Biomed Clear Resin, Formlabs, Somerville, MA, USA) and was manufactured using a stereolithography printer (Form 3B, Formlabs, Somerville, MA, USA). Each tooth was placed and bonded into a socket, which was held by flexible horizontal bars. The bar dimensions (width: 3 mm; height: 1.7 mm) and the vertical distance between the bars (2.6 mm) were adjusted to allow physiological tooth mobility. Tooth mobility was tested in all models (three teeth per model) before the main experiments began and all models had comparable tooth mobility to that of the clinical situation [28]: horizontal tooth mobility: 0.36 ± 0.06 mm/100 N; axial tooth mobility: 0.11 ± 0.01 mm/100 N. We also tested whether the models could withstand cyclic mastication forces (1,200,000 cycles, 60 N) and maximum mastication forces above 500 N, which exceeds the physiological mastication forces in the incisal area of about 230 N [29]. For every retainer, eight in vitro tooth models (56 models in total) were produced and tested.

### Building the sample

**Fig. 2:**
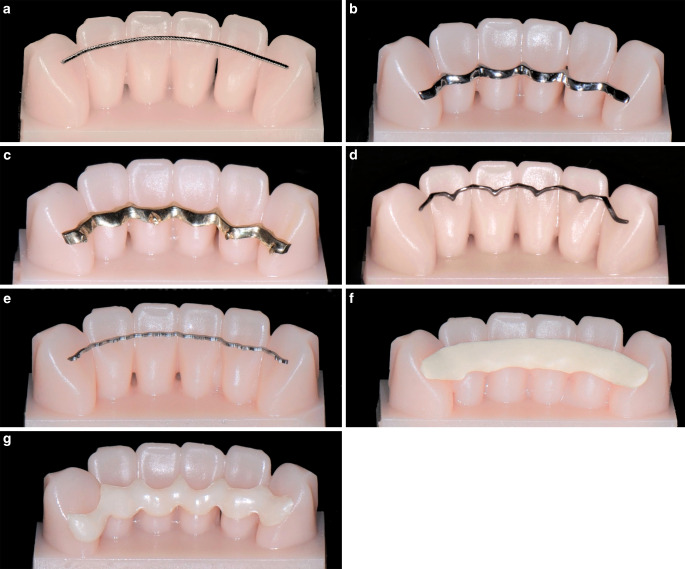
Tested retainers—conventional twistflex retainers (**a**) and six commercially available computer-aided design/computer-aided manufacturing (CAD/CAM) retainers (**b**–**f**) were tested. The CAD/CAM retainers were made from cobalt–chromium (**b**), gold (**c**), titanium grade 5 (**d**), nickel–titanium (**e**), polyetheretherketone (**f**) and zirconia (**g**) Die getesteten Retainer – herkömmliche Twistflex-Retainer (**a**) und 6 kommerziell erhältliche CAD/CAM(„computer-aided design/computer-aided manufacturing“)-Retainer (**b**–**f**). Die CAD/CAM-Retainer waren aus Kobalt-Chrom (**b**), Gold (**c**), Titan Grad 5 (**d**), Nickel-Titan (**e**), Polyetheretherketon (**f**) und Zirkoniumdioxid (**g**) gefertigt

The twistflex retainers were bent by hand onto each in vitro model. For the CAD/CAM retainers, all in vitro tooth models were scanned with an intraoral scanner (Trios 4, 3Shape, Copenhagen, Hovedstaden, Denmark) and a separate standard tessellation language (STL) file was generated for each model. The STL files were sent to the manufacturers. Eight CAD/CAM retainers were made from each of the six materials. These materials were cobalt–chromium (CoCr), gold, titanium grade 5 (Ti5), nickel–titanium (NiTi), polyetheretherketone (PEEK) and zirconia (ZrO_2_) (Table 1, Fig. 2).

**Table 1:** Details of tested retainers Details der getesteten Retainer

Material (product name)	Material composition (%)	Manufacturer	Production technique	Primer on tooth	Adhesive
Stainless steel (Respond archwire; Twistflex)	Fe = main component; C ≤ 0.08; Cr ≤ 18–20; Ni ≤ 8–10.5; Mn ≤ 2; silicon ≤ 1	Ormco, CA, USA	Bending	Ceraresin Bond (for all)	Transbond XT (for all except Zirconia)
Cobalt–chromium (No specific product name)	Co ≤ 60.5; Cr ≤ 28; W ≤ 9; Si ≤ 1.5	Team Ziereis, Engelbrand, Germany	Laser melting		
Gold alloy (No specific product name)	Au ≤ 73.8; Ag ≤ 9.2; Pt ≤ 9.0; Cu ≤ 4.4; Zn ≤ 2.0; In ≤ 1.5; Ir ≤ 0.1	Team Ziereis, Engelbrand, Germany	Milling		
Titanium grade 5, Ti6Al4V (3D Titan Retainer)	Ti = main component; Al ≤ 5.5–6.75; V ≤ 3.5–4.5; Fe, O, N, C, H: all ≤ 1	Hochstetter Dental/Klee, Frankfurt, Germany	Milling		
Nickel–titanium (Memotain)	Ni ≤ 55; Ti ≤ 45; O, N, C: all ≤ 1	CA-Digital, Hilden, Germany	Laser cutting		
Polyetheretherketone (PEEK) (No specific product name)	PEEK ≤ 80; TiO_2_ ≤ 20; TiO_2_ based pigment ≤ 1; Fe_2_O_3_ ≤ 1	Eutiner Zahntechnik, Eutin, Germany	Milling		
Zirconia (No specific product name)	ZrO_2_ = main component; Y_2_O_3_ ≤ 4–6; Al_2_O_3_ ≤ 1; SiO_2_, Fe_2_O_3_, Na_2_O: all ≤ 1	Zahnwerkstatt, Wernigerode, Germany	Milling		Panavia V5 (for Zirconia)

Before the retainers were bonded to the models, all model teeth were sandblasted (50 μm alumina particles, 1 bar) and conditioned with a primer (Ceraresin Bond 1&2, Shofu, Tokyo, Japan) as previously described [26]. NiTi, Ti5, gold, PEEK, and twistflex retainers were bonded with composite (Transbond XT, 3M, Saint Paul, MN, USA) and light cured with a dental light curing device (460 nm; Smartlite focus; Dentsply Sirona, Charlotte, NC, USA) for 40 s on every tooth according to previous studies [26, 30]. ZrO_2_ retainers were prepared with tribochemical silica coating using Rocatec (RC; 3M ESPE; Seefeld, Germany) on the bonding site: RC Pre (lot 467012, 0.28 MPa, distance: 10 mm, duration: 10 s, angle: 45°) and RC Plus (lot 432661, 0.28 MPa, distance: 10 mm, duration: 13 s, angle: 45°). Afterwards, the primer (Clearfil Ceramic Primer Plus, Kuraray Noritake, Tokyo, Japan) and the composite cement (Panavia V5, Kuraray Noritake, Tokyo, Japan) were successively applied onto the ZrO_2_ retainers. ZrO_2_ retainers were placed onto the corresponding model and light cured for 40 s (Smartlite focus, Dentsply Sirona, Charlotte, NC, USA) from four directions on every tooth as previously described [31] and excess cement was removed. The whole bonding procedure was performed by the same dentist in accordance with the manufacturers’ instructions.

### Ageing and load capacity testing

**Fig. 3:**
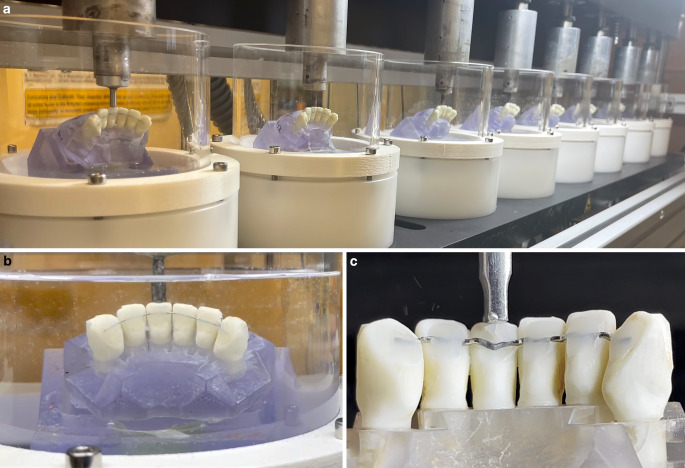
Testing protocol: to simulate 15 years of wearing time, all retainer models were loaded 1,200,000 times in a chewing simulator in water (for better visibility photo in **a** was taken without water in chambers) (**a**, **b**). Retainers that were not damaged by the ageing process were further loaded in a universal testing device until retainer debonding or breakage was detected (**c**) Prüfprotokoll: Um eine Tragezeit von 15 Jahren zu simulieren, wurden alle Retainermodelle 1.200.000-mal in einem Kausimulator in Wasser belastet (zur besseren Sichtbarkeit wurde das Foto in **a** ohne Wasser in den Kammern aufgenommen) (**a**, **b**). Retainer, die durch den Alterungsprozess nicht beschädigt wurden, wurden daraufhin in einem Universalprüfgerät weiter belastet, bis ein Klebeversagen oder ein Bruch festgestellt wurde (**c**)

After bonding, all retainer models underwent the following standardised ageing process (Fig. 3a,b):

- 1,200,000 chewing cycles (force magnitude 65 N, loading direction tilted by 45° to the vertical axis; CS‑4, SD Mechatronik, Feldkirchen-Westerham, Germany) were exerted on tooth 31. During chewing simulation, the model was immersed in water at room temperature (23 ± 1 °C).
- Water storage for 30 days at body temperature (37 ± 1 °C).

After the ageing process, the models were checked for failures, i.e. retainer fracture or debonding. In retainer models that did not fracture or debond during ageing, the fracture resistance F_max_ of tooth 31 was tested in a universal testing machine (Zwick, Roell, Ulm, Germany; Fig. 3c). High loads generally correspond to axial forces, so the load was applied on tooth 31 with a steel piston in a vertical direction. The crosshead was lowered with a speed of 2 mm/min until a drop-in test force ≥ 20% of the maximum test force occurred.

### Statistical evaluation

Statistical analysis was performed using SPSS statistics 27 (IBM, Armonk, NY, USA). F_max_ values were compared between the different groups of retainers. In line with previous studies and because of statistical reasons [32, 33], retainers that failed during the ageing process were associated with a load capacity of F_max_ = 65 N. Data were analysed using Kruskal–Wallis tests first in order to find general effects. Afterwards pairwise Mann–Whitney U‑tests were used to compare twistflex retainers to the individual CAD/CAM retainers. Multiple testing was excluded by using Bonferroni correction. The significance level was set to *p* = 0.05.

## Results

**Fig. 4:**
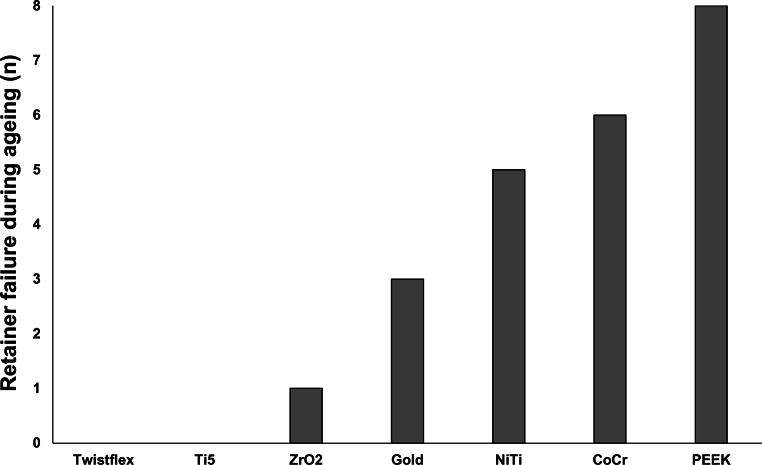
Number of retainer failures during ageing—Twistflex retainers and computer-aided design/computer-aided manufacturing (CAD/CAM) titanium grade 5 retainers were the only retainers which did not fail during the ageing process. Of the CAD/CAM retainers that failed, ZrO_2_ retainers had the lowest failure rate and polyetheretherketone (PEEK) retainers had the highest failure rate Anzahl der Retainerausfälle während der Alterung – Twistflex-Retainer und CAD/CAM(„computer-aided design/computer-aided manufacturing“)-Retainer aus Titan Grad 5 waren die einzigen Retainer, die während des Alterungsprozesses nicht versagten. Unter den anderen CAD/CAM-Retainern zeigten ZrO_2_-CAD/CAM-Retainer die geringsten und PEEK(Polyetheretherketon)-Retainer die höchsten Versagensraten

**Fig. 5:**
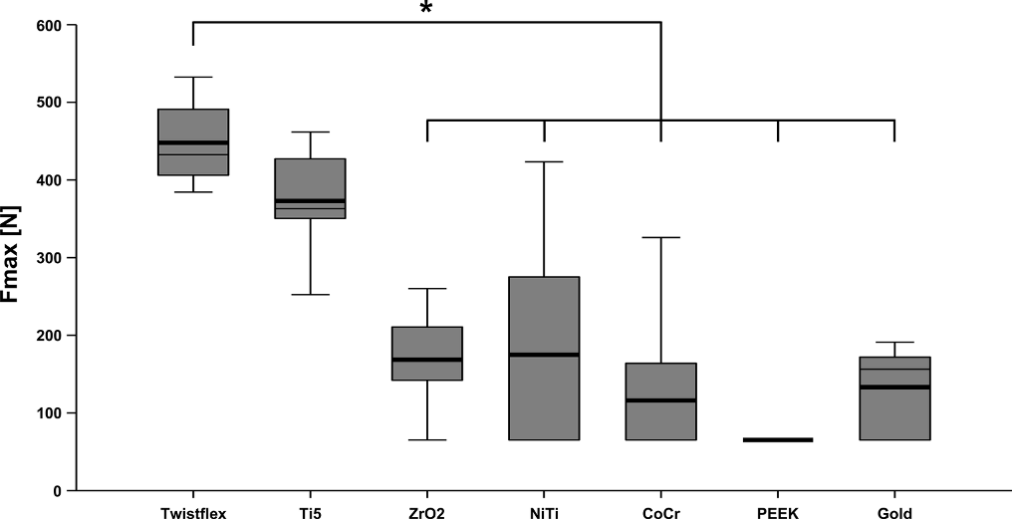
Load capacity (F_max_) testing—Twistflex retainers had the highest F_max_ values. Titanium grade 5 retainers were the only computer-aided design/computer-aided manufacturing (CAD/CAM) retainers with a comparable F_max_ to twistflex retainers. All other CAD/CAM retainers had significantly lower F_max_ values (*). Retainers that failed during the ageing process were associated with a load capacity of F_max_ = 65 N. Prüfung der Maximalbelastung (F_max_) – Twistflex-Retainer zeigten die größten F_max_-Werte. Retainer aus Titan Grad 5 zeigten als einzige CAD/CAM(„computer-aided design/computer-aided manufacturing“)-Retainer vergleichbare F_max_-Werte zu Twistflex-Retainern. Alle anderen CAD/CAM-Retainer zeigten signifikant geringere F_max_-Werte (*). Retainer, die während des Alterungsprozesses versagten, wurde eine F_max_ von 65 N zugewiesen

Twistflex retainers did not fail during the ageing process and had the highest F_max_ values (445.8 ± 51.2 N; Figs. 4 and 5). Ti5 retainers were the only CAD/CAM retainers that also did not fail during ageing and had similar F_max_ values to twistflex retainers (374.0 ± 62.4 N). All other CAD/CAM retainers failed to varying extents during ageing and had significantly lower F_max_ values than twistflex retainers did (*p* < 0.01). Of these failed retainers, ZrO_2_ retainers had the lowest failure rates (1/8; F_max_ = 168.8 ± 52.4 N) followed by gold retainers (3/8; F_max_ = 130.2 ± 51.8 N), NiTi retainers (5/8; F_max_ = 162.2 ± 132.6 N), CoCr retainers (6/8; F_max_ = 122.27 ± 100.5 N) and PEEK retainers (8/8; F_max_ = 65 ± 0 N).

**Fig. 6:**
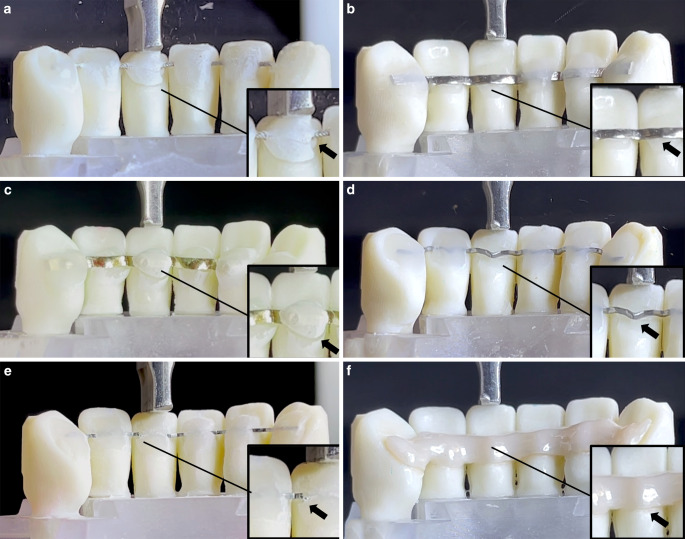
Event of failure during load capacity (F_max_) testing—Twistflex (**a**), cobalt–chromium (**b**), gold (**c**), titanium grade 5 (**d**), and zirconia (**f**) computer-aided design/computer-aided manufacturing (CAD/CAM) retainers failed because of debonding. NiTi retainers (**e**) were the only CAD/CAM retainers which failed because of breakage or debonding. Polyetheretherketone (PEEK) CAD/CAM retainers were not tested for F_max_, since all failed during ageing Moment des Versagens unter Maximalbelastung (F_max_) – Twistflex (**a**), Kobalt-Chrom (**b**), Gold (**c**), Titan Grad 5 (**d**) und Zirkoniumdioxid (**f**) CAD/CAM(„computer-aided design/computer-aided manufacturing”)-Retainer versagten alle aufgrund von Debonding. NiTi-Retainer (**e**) waren die einzigen CAD/CAM-Retainer, die neben einem Debonding auch Brüche zeigten. PEEK(Polyetheretherketon)-CAD/CAM-Retainer wurden nicht auf F_max_ getestet, da sie alle bereits während des Alterungsprozesses durch Debonding versagten

The CAD/CAM retainers failed because of debonding during ageing and F_max_ testing, except for the NiTi retainers, which failed because of breakage or debonding (5/8 broke during ageing, 1/8 broke during F_max_ testing and 2/8 debonded during F_max_ testing). The moment of failure is illustrated in Fig. 6.

## Discussion

The null hypothesis had to be rejected because conventional five-stranded stainless steel twistflex retainers showed the highest F_max_ values and demonstrated no failure during simulated ageing. Therefore, conventional five-stranded stainless steel twistflex retainers can still be considered as the gold standard, which is in line with the recommendation by Zachrisson et al., which was based on their 20 years of experience with multistranded retainer wires [34]. We showed that most CAD/CAM retainers (except for Ti5 retainers) presented higher failure rates during ageing and significantly lower F_max_ values (*p* < 0.01) than twistflex retainers. Based on these findings, only Ti5 CAD/CAM retainers can be considered a valid alternative to conventional hand-bent twistflex retainers.

There are several methodological strengths to this study. First, to the best of our knowledge, this is the first study to investigate the survival of multiple CAD/CAM retainers and compare these survival rates with those of the conventional twistflex retainer. Previous studies have only investigated NiTi [17, 19, 22, 23] or PEEK CAD/CAM retainers [15] and other studies were individual case reports presenting single CAD/CAM retainers made from PEEK [14], NiTi [16] or ZrO_2_ [20]. In contrast, we tested a representative sample of six different CAD/CAM retainers with standardised experimental procedures and reliable in vitro comparisons. Because of the reliability of our experimental setup, we decided not to compare these six CAD/CAM retainers in a clinical trial. Long-term clinical trials often suffer from limited standardisation, including factors like differences in chewing behaviour/forces or differences in treatment prior to retention. In addition, informative results from clinical trials are only possible after a long observation time.

Another strength of our methodology is that in vitro tooth models were specifically developed for this study and tooth mobility was tested in every model before the main investigation started. These tests confirmed the physiological mobility of the model teeth, showing that the model was valid for clinical simulation. The model could also test the retainers in their whole geometry, like in the patient. Previous studies have not taken into account physiological tooth mobility [35–39] and have not tested the whole geometry of the retainers but rather just on one [25], two [35–37] or three teeth [39]. However, not considering the whole geometry of the retainer and the physiological mobility of the teeth can affect the biomechanical behaviour of the retainer and produce misleading results. These earlier studies may have been limited by the availability of extracted human teeth. We avoided this limitation by investigating alternatives to human teeth in a previous study [26] and found FRC an ideal material for the production of teeth for in vitro testing because its bonding strength (18.0 ± 2.4 MPa) was comparable to that of the clinical situation [27]. Using CAD/CAM teeth also allowed us to use the same geometries for all models, ensuring standardised experimental conditions.

To the best of our knowledge, this is the first study to use a chewing simulator to investigate orthodontic devices. Therefore, we were able to simulate 15 years of wearing time, which tells us far more about the long-term survival of retainers than previous studies have, with follow-up times limited to 1 year [17] or 6 months [19, 22, 23]. This longer observation time might also explain why previous studies did not detect differences in the survival rates between twistflex and CAD/CAM NiTi retainers. Using our highly standardised in vitro model, we showed that, except for Ti5 retainers, CAD/CAM retainers result in higher long-term failure rates than twistflex retainers, which within our study, showed no failure at all.

Our study revealed that NiTi CAD/CAM retainers are more prone to breakage than other CAD/CAM retainers. This has important implications in the clinical situation because NiTi CAD/CAM retainers may need to be removed instead of just rebonded after breakage resulting in increasing costs and burden for the patient and time and effort for the practitioner. Also, the low F_max_ values of CAD/CAM retainers might have clinical consequences. This is because the F_max_ values in all tested CAD/CAM retainers (except for Ti5 retainers) were lower than the maximal incisor bite force of about 200 N [40–42]. In ZrO_2_ retainers, these low F_max_ values seem to make them less suitable for long-term retention, although they were largely resistant to ageing.

Of note, debonding and breakage were not visually detectable during the initial chewing simulation but rather later on during the F_max_ testing. For the detachment of ZrO_2_, CoCr, PEEK and gold retainers, this might have been caused by the stiffness and bulkiness of these retainers, which prevented visual inspection of the bonding area. In NiTi retainers, breaking during ageing was undetected because of their high springback properties, which returned the retainer to its original form as soon as the load was released. Undetected debonding and breakage in CAD/CAM retainers may be problematic because the patient may not notice the failure until the teeth begin to move. The orthodontist will also not notice the failure if the orthodontic therapy was finalised years ago and regular control visits have already been discontinued.

Finally, when interpreting the results of the present study, it has to be considered that although our model aimed to simulate the clinical situation as closely as possible, this was still an in vitro study, so drawing specific clinical conclusions is limited. Although the CAD/CAM teeth used in the present study were validated for having similar bonding strength values as compared to human and bovine teeth [26], there were still slight differences with respect to the adhesive remnant index. Second, we tested CAD/CAM retainers that are commercially available to give our readers the information they need to protect their patients from unnecessary burdens. This is why we refrained including own design preferences to the respective manufacturers. However, modifying the design or using other materials for the production might affect the biomechanical behaviour and therefore the survival of CAD/CAM retainers, which should be investigated in future studies.

## Conclusions

The results of the present in vitro study have shown that

- Twistflex retainers showed no failure during the ageing process and demonstrated the highest maximum load capacity of all retainers tested. Therefore, twistflex retainers should remain the gold standard for long-term sufficiency.
- Ti5 retainers were the only computer-aided design/computer-aided manufacturing (CAD/CAM) retainers that did not fail during the ageing process and had similar load capacity values to the twistflex retainer. Therefore, Ti5 CAD/CAM retainers may represent a suitable alternative.
- NiTi, ZrO_2_, gold, CoCr and polyetheretherketone (PEEK) CAD/CAM retainers all failed during ageing and had significantly lower F_max_ values than twistflex retainers; therefore, the long-term sufficiency of these retainers may be limited.
